# Despite its sequence identity with canonical H4, *Drosophila H4r* product is enriched at specific chromatin regions

**DOI:** 10.1038/s41598-022-09026-x

**Published:** 2022-03-23

**Authors:** Andrea Ábrahám, Zoltán Villányi, Nóra Zsindely, Gábor Nagy, Áron Szabó, László Bodai, László Henn, Imre M. Boros

**Affiliations:** 1 Institute of Biochemistry, Biological Research Centre of Szeged, Szeged, 6726 Hungary; 2 Department of Biochemistry and Molecular Biology, Faculty of Science and Informatics, University of Szeged, Szeged, 6726 Hungary; 3 Doctoral School in Biology, Faculty of Science and Informatics, University of Szeged, Szeged, 6726 Hungary; 4 Department of Genetics, Faculty of Science and Informatics, University of Szeged, Szeged, 6726 Hungary; 5 Institute of Genetics, Biological Research Centre of Szeged, Szeged, 6726 Hungary

**Keywords:** Histone variants, Drosophila

## Abstract

Histone variants are different from their canonical counterparts in structure and are encoded by solitary genes with unique regulation to fulfill tissue or differentiation specific functions. A single H4 variant gene (*His4r* or *H4r*) that is located outside of the histone cluster and gives rise to a polyA tailed messenger RNA via replication-independent expression is preserved in *Drosophila* strains despite that its protein product is identical with canonical H4. In order to reveal information on the possible role of this alternative H4 we epitope tagged endogenous H4r and studied its spatial and temporal expression, and revealed its genome-wide localization to chromatin at the nucleosomal level. RNA and immunohistochemistry analysis of *H4r* expressed under its cognate regulation indicate expression of the gene throughout zygotic and larval development and presence of the protein product is evident already in the pronuclei of fertilized eggs. In the developing nervous system a slight disequibrium in H4r distribution is observable, cholinergic neurons are the most abundant among H4r-expressing cells. ChIP-seq experiments revealed H4r association with regulatory regions of genes involved in cellular stress response. The data presented here indicate that H4r has a variant histone function.

## Introduction

The *Histone 4 replacement* gene *(H4r)* encodes a protein identical in amino acid sequence with its canonical histone H4 counterpart. *H4r* has been identified in 14 out of 22 sequenced *Drosophila* species so far^1^. Unlike canonical *H4* genes, which are found in multiple copies within the histone cluster on the second chromosome^2^, *H4r*, similarly to the other histone variants, is located outside of the canonical histone cluster in a single copy. The third chromosomal *H4r* gene contains an intron, expressed independently of replication and produces polyadenylated mRNA product^2^. *H4r* mRNA is only weakly expressed in the germline and shows much higher expression in terminally differentiated cells. This observation led to the hypothesis on H4r replaces canonical H4 in postmitotic cells due to its replication independent expression^2^. Another hypothesis on the role of the alternative H4 is that it may play a role in environmental stress response, as mRNA expression from *H4r* changes upon ethanol treatment^3^. Since *H4r* encodes the same amino acid sequence as the canonical *H4* in 14 *Drosophila* species with a codon usage different from that of *H4*, a further theory for the function of H4r is that it is incorporated to the chromatin with distinct co- or post-translational modifications in distinct cell types or in different environmental conditions^1^. Deletion of *H4r* does not cause a visible phenotypic change^4^, however, the loss of *H4r* causes reduced viability; female *H4r* mutants showing lower viability than males. Loss of *H4r* is also coupled to increased heat-stress resistance, supposedly due to the less condensed chromatin that allows a quicker and stronger response to heat stress^5^.

Although little data are available on the expression of *H4r*, on NCBI and on FlyBase it is indicated that the amount of H4r may differ significantly in distinct cell types of a tissue. For instance, experiments performed on larval and adult brains at various stages show that the amount of repressive marker PcG increases on *H4r* by the progression of differentiation whereas the amount of RNA polymerase II decreases, suggesting that *H4r* is expressed in undifferentiated cells, but not or at a lower level in mature neurons^6^. There are currently no data available in the literature about the genomic distribution and interaction partners of H4r. Intrigued by the fact that a replacement histone gene with a gene product of identical structure but different expression pattern as the corresponding canonical histone is preserved in *Drosophila* species, we performed a detailed analysis of *H4r* expression and H4r localization in order to gain information on the function of this so far enigmatic gene and its product.

## Results

### Tagging H4r with 3xFlag-tag

To overcome the problem that the amino acid sequences of the products of *H4r* and *H4* are identical making the two proteins indistinguishable, we fused a 3xFlag epitope tag encoding DNA sequence to the genomic *H4r* gene retaining its original expression pattern (Supplementary Figure S1). As a result, with the use of a Flag-specific antibody the product of *H4r* can be detected among the structurally identical H4 proteins. We created fly strains in which the 3xFlag-tag is located at the N-terminus of H4r and analysed it from a developmental, cellular and molecular perspective. To facilitate identification of labelled H4r animals, we included a dsRed marker gene flanked by two loxP recombination sites downstream of H4r. Because expression of the red fluorescent marker protein dsRed in brain interferes with analysis of images of immunostained brains, it was necessary to remove this marker gene before sample preparation. We achieved this by Cre-mediated recombination.

### Analysis of H4r expression

**Figure 1:**
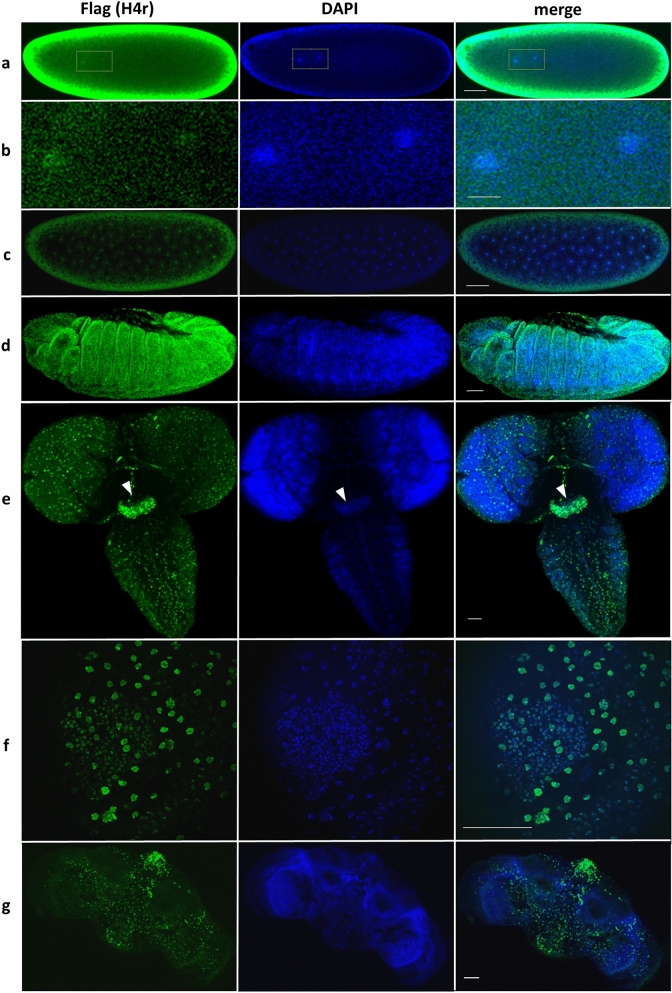
Expression of H4r in embryo and brains of larvae and adults. (**a**) Fertilized embryo; Framed inlet highlights pronuclei; (**b**) Pronuclei; (**c**) Early embryo (nc 7); (**d**) Gastrulating embryo; (**e**) Larval brain (eye discs and ventral nerve cord, arrowhead: suboesophagial ganglion); (**f**) Eye disc; (**g**) Adult brain. Scalebars refer to 50 µm on (**a, c**), (**d-g**), 10 µm on (**b**).

Taking advantage of the Flag epitope tagged H4r expressed under its canonical control, we performed immunohistochemical stainings to detect H4r presence in different stages of development in different tissues and cell types. We found that H4r was detectable in both male and female pronuclei of embryos, and the expression of H4r remained ubiquitous throughout embryonic development (Fig. 1a–d). In the brain of wandering larvae and adults different levels of H4r expression was observed (Fig. 1e–g). Anti-Flag and DAPI double staining did not show clearly observable differences in the chromatin compaction of the Flag-positive cells. In order to identify cell types expressing H4r in larval brain we generated transgenic lines in which cell type-specific Lamin B-GFP expression could be achieved using the Gal4-UAS system. For the *Drosophila* stocks used for this experiment see Supplementary Table S3. By using these lines we sought to identify cell types in which colocalization of H4r with Lamin B-GFP could be detected.

**Figure 2:**
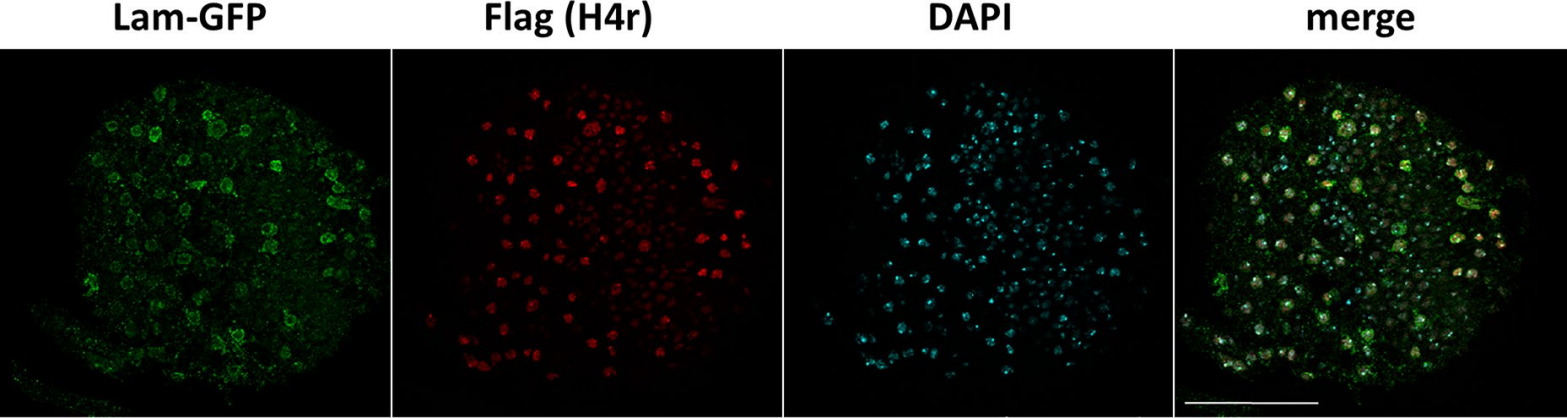
Colocalization between cholinergic neurons (green) and H4r expressing cells (red) in the larval brain (eye disc). 69.2% of H4r-expressing cells are cholinergic neurons. Scalebar refers to 50 µm.

Analysing expression by immunohistochemistry in *Drosophila* lines expressing 3xFlag-H4r and Lamin B-GFP under the control of *OK371, Gad1, ChAT, elav* and *Insc* permitting the identification of glutaminergic, GABAergic, cholinergic neurons, mature neurons in general and neuroblasts, respectively, we found partial colocalization of lamin and Flag positive nuclei in every *Drosophila* line. This observation indicates that the expression of H4r is not restricted strictly and characteristic uniquely for any of the investigated neuron types. Nonetheless, we found that although the colocalization was not perfect, the majority (53.38% of cells in the whole brain, 69.2% in the eye discs) of H4r accumulating cells was cholinergic neurons (Fig. 2). Weak level of colocalization were found with mature neurons in general, with glutaminergic and GABAergic neurons and with neuroblasts (Table 1 and Supplementary Figure. S2).

**Table 1:** Major cell types accumulating H4r in the larval brain. Colocalisation rate represents % of H4r-expressing cells belonging to the driver-indicated type of neuron.

Genotype of line created in this study	Types of cells expressing LamB-GFP	Colocalisation rate (%)
*w; OK371-Gal4/UAS-LamB-GFP; 3xFlag-H4r*	Glutaminergic neurons^25,26^	11.52
*w; Gad1-Gal4/UAS-LamB-GFP; 3xFlag-H4r*	GABAergic neurons^26^	19.08
*w; ChaT-Gal4/UAS-LamB-GFP; 3xFlag-H4r*	Cholinergic neurons^26,27^	53.38
*w; Insc-Gal4/UAS-LamB-GFP; 3xFlag-H4r*	Neuroblasts^28^	16.39
*elav-Gal4/w;UAS-LamB-GFP/*+*; 3xFlag-H4r/*+ *elav-Gal4/Y;UAS-LamB-GFP/*+*; 3xFlag-H4r/*+	Mature neurons^29^	41.74

### The expression of H4r does not change significantly upon heat stress

**Figure 3:**
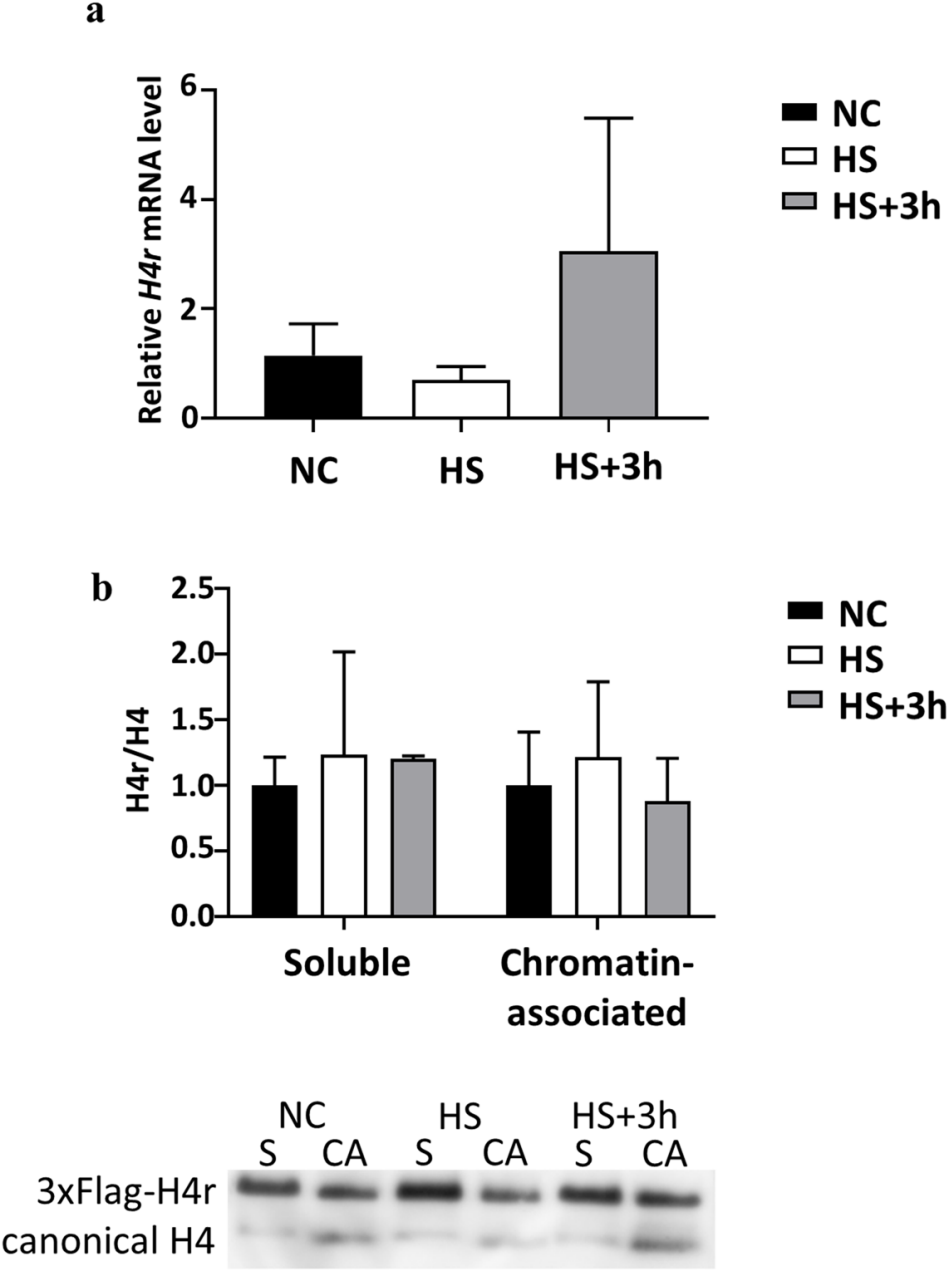
No significant changes can be detected in the expression and in the amount of H4r incorporated to chromatin upon heat shock and recovery. (**a**) Changes in the expression of *H4r* mRNA upon heat shock and recovery. (normalised to *tubulin*; n = 2; p ≥ 0.1357; one-way ANOVA.); (**b**) Changes in the amount of soluble and chromatin associated H4r upon heat shock and recovery. NC: negative control; HS: heat shocked; HS + 3 h: heat shocked and relaxed (at room temperature) for three hours. S: soluble, CA: chromatin associated (n = 2; p ≥ 0.7529, one-way ANOVA. Error bars represent s.d.).

H4r was suggested to play a role in the chromatin formation at the loci of inducible genes such as heat shock genes^5^. We wondered whether there are changes in *H4r* expression upon heat shock and following recovery, which would further clarify the role of H4r in the expression of inducible genes. Therefore, we measured the changes in *H4r* expression at mRNA and protein levels upon heat stress and recovery. In addition we also determined if change in the ratio of soluble and chromatin associated H4r was detectable under the above conditions. We found no significant change in *H4r* expression at either mRNA (normalised to *tubulin*; n = 2; p ≥ 0.1357; one-way ANOVA with Sidak’s multiple comparison test) or protein level, nor did we find alteration in the distribution of H4r protein between free (n = 2; p ≥ 0.8675; two-way ANOVA with Tukey’s multiple comparison test) and chromatin associated forms (n = 2; p ≥ 0.7529; two-way ANOVA with Tukey’s multiple comparison test) (Fig. 3).

### H4r preferentially binds to specific chromosomal loci

**Figure 4:**
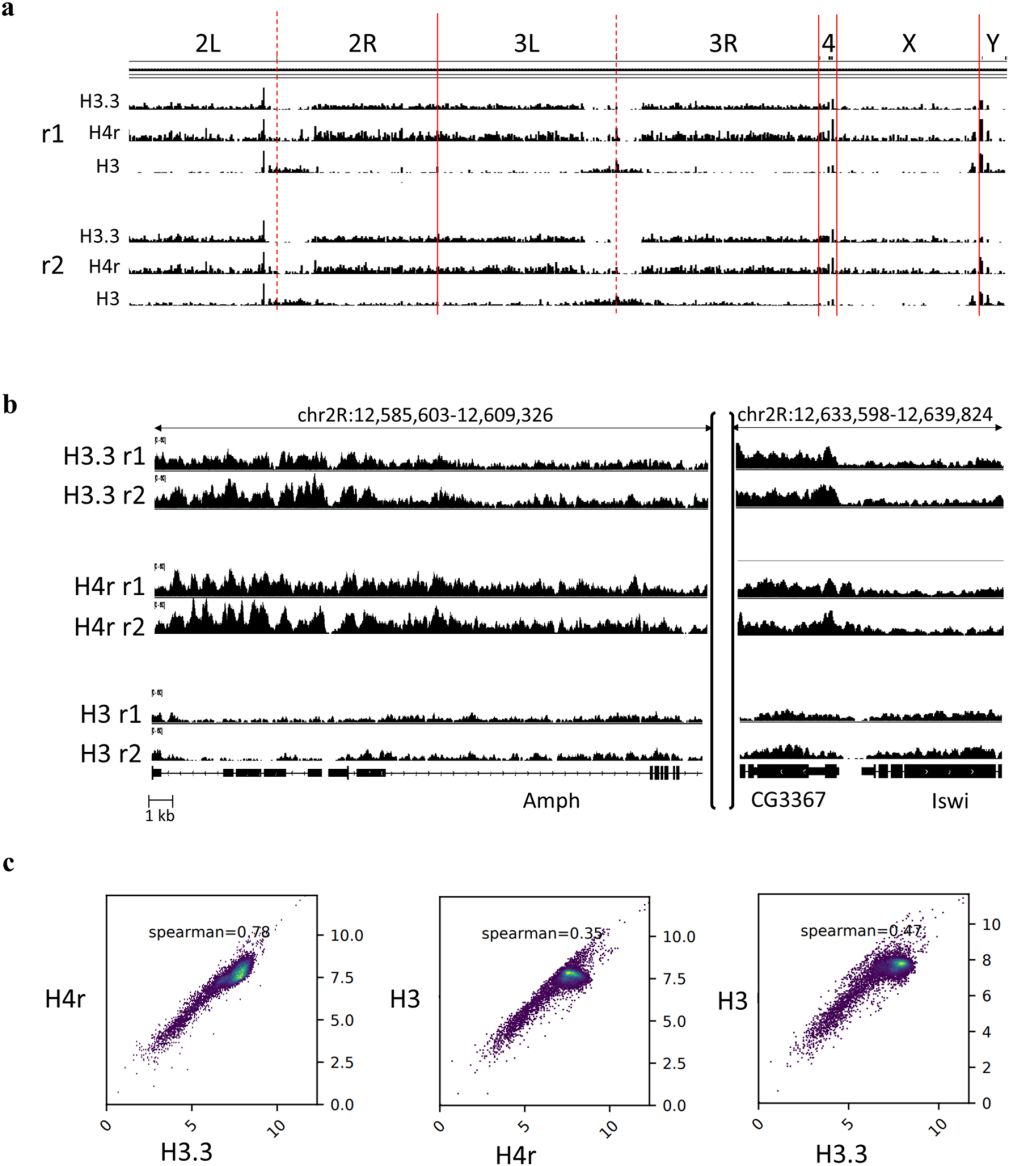
Comparison of genome-wide distribution of H4r, H3.3 and H3. (**a**) Genome-wide distribution of H3.3, H4r and H3 as determined by replicates of ChIP-seq experiments. On the top the extensions of individual chromosomes are shown, the graphs indicate the detected frequencies of localization distribution of the indicated proteins. Note that H3.3 and H4r were both detected by Flag specific antibody while H3 was detected a specific antibody, which also recognise H3.3. (**b**) H3.3, H4r and H3 localization at specific genome regions. r1: replicate 1, r2: replicate 2. (**c**) Spearman correlation scatterplots of H4r, H3.3 and H3 ChIP-seq enrichment.

One way of obtaining hints on H4r specific function could be the determination of its association with loci of particular chromosomal regions or genome-wide. Staining of polytene chromosomes gave no information in this respect since H4r seemed to be associated with regions of giant chromosomes throughout roughly in inverse proportion of RNA polymerase II. In order to examine genomic distribution of H4r in diploid cells with a better resolution we performed chromatin-immunoprecipitation followed by sequencing (ChIP-seq) assays for epitope tagged H4r with Flag antibody. In parallel we also detected H3 distribution. Our aim was to detect the genome-wide nucleosome occupancy and analyze H4r distribution. As H3 is present in nucleosomes with H4 in equimolar quantity the two can provide identical information about nucleosome occupancy. Therefore instead of H4 antibody, we used an H3-specific antibody in these experiments as the latter one gave more consistent results in ChIP assays. In addition, we also detected the localization of the H3 variant H3.3. To achieve this, for chromatin preparation we used a *Drosophila* line which expresses transgenic H3.3 fused with a 3xFlag tag under the control of elav-Gal4. Thus, as the H3-antibody recognises both the canonical H3 and H3.3 but the Flag-antibody only H3.3, we could determine — using the same samples — H3 localization reflecting chromatin compaction and also H3.3 distribution. We found extensive similarities between H4r and H3.3 localizations and significant differences in the localization of these variants and that of canonical H3 (Fig. 4a).

Results of repeated ChIP-seq experiments indicated a reproducible non-random and non-uniform localization of H4r throughout the genome (Fig. 4b). Overall, H4r and H3.3 shared extensive similarity in their genome-wide distribution (Spearman’s rank correlation coefficient ρ = 0.78, p = 0.0041, Fig. 4c). However, the overlap between the localization of the two histone variants was far from being perfect, suggesting functional differences for the two alternative histone forms.

**Figure 5:**
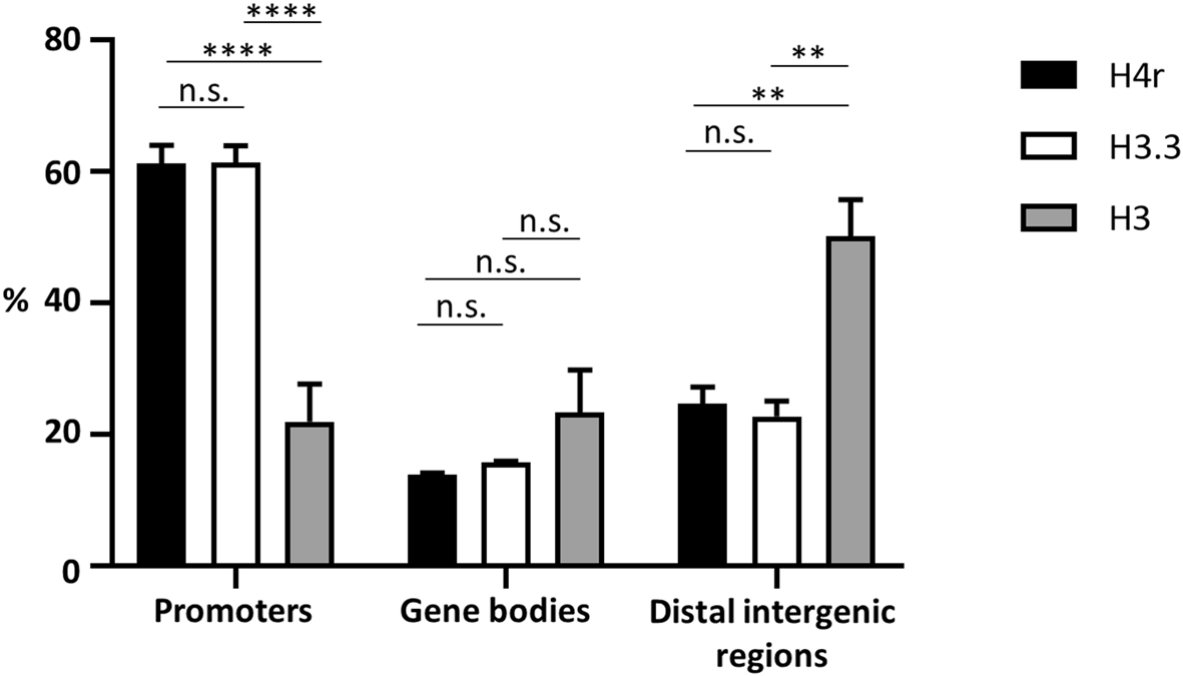
Distribution of H4r, H3.3 and canonical H3 on different genomic regions. Promoters: TSS ± 1 kb; Gene bodies: 5’UTR, exons, introns, 3’UTR, downstream 300 bp; Distal intergenic regions: > 300 bp downstream to 3’-end of genes. Statistical analysis method used: Tukey-test (n = 2; n.s.: not significant; *: p < 0.05; **: p < 0.01; ***: p < 0.001; ****: p < 0.0001. Error bars represent s.d.).

Notably, H4r distribution did not show strong correlation with that of H3, which can be assumed to reflect canonical H4 distribution (Spearman’s rank correlation coefficient ρ = 0.35, p = 0.0187, see Fig. 4c), but was similar to H3.3 (as it was expected, the correlation between H3.3 and canonical H3 was higher, Spearman’s rank correlation coefficient ρ = 0.47, p = 0.1695, see Fig. 4c). Identification of the regions at which H4r was specifically found in higher abundance revealed that this variant histone was most frequently bound to promoter regions (61.3% of total H4r were found at promoter regions, 14.0% at gene bodies and 24.7% at distal intergenic regions; 61.4% of total H3.3 were found at promoter regions, 15.8% at gene bodies and 22.8% at distal intergenic regions). On the contrary, a lower enrichment of H4r was detected in intergenic regions compared to the canonical H3 (22.0% of total H3 were found at promoter regions, 27.9% at gene bodies and 50.1% at distal intergenic regions) (Fig. 5). Altogether, these results indicated H4r to be more similar functionally to a histone variant, such as H3.3 than to a canonical histone.

Next, we analyzed which functional groups of genes show H4r enrichment. According to a PANTHER GO-Slim Biological Process analysis H4r, similarly to H3.3, shows enrichment on genes coupled with differentiation or necessary for normal functions – including genes inducible by various stimuli – and did not show significant enrichment on genes associated with cell cycle and cell division (Supplementary Table S1).

**Figure 6:**
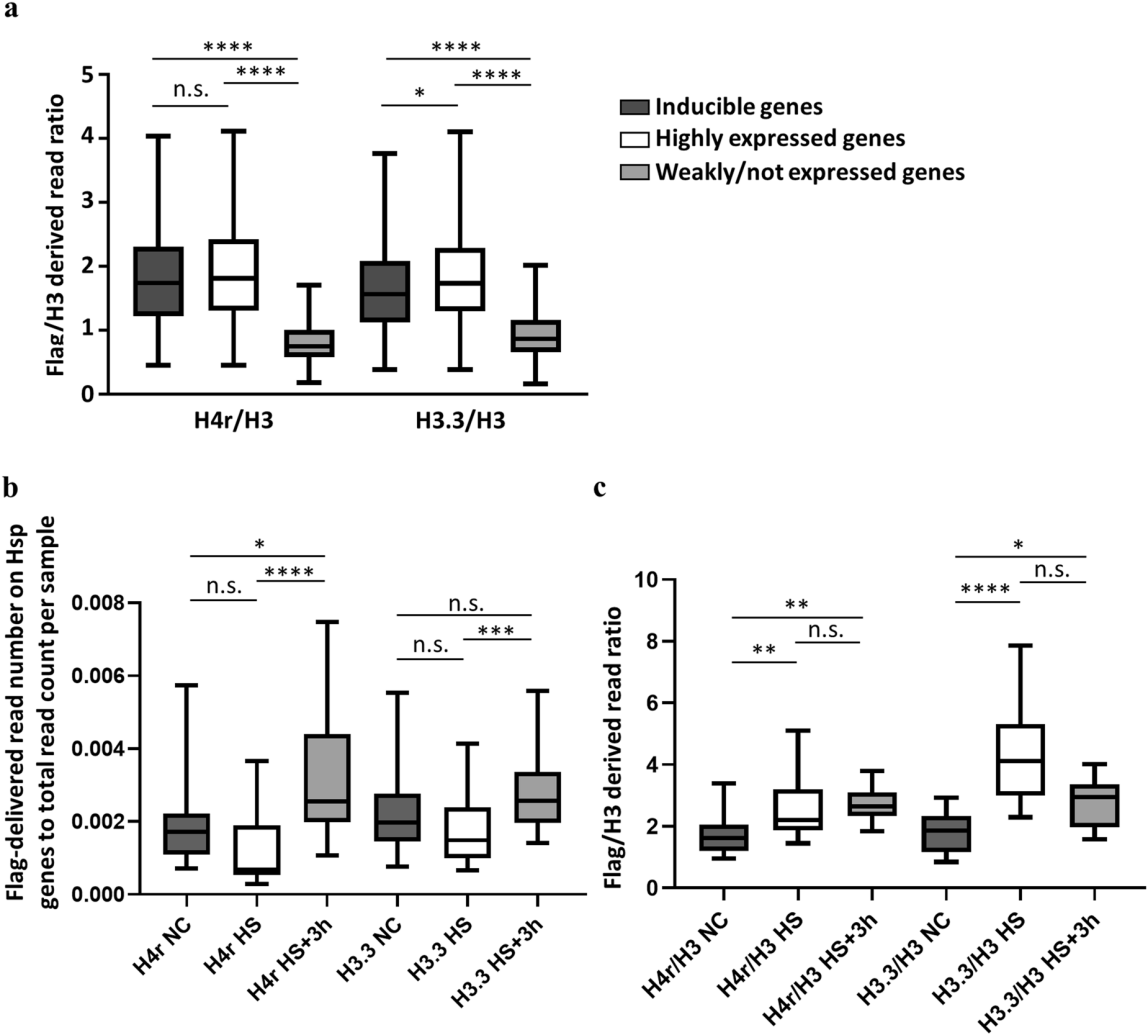
H4r and H3.3 nucleosomal distribution on inducible genes. (**a**) Amount of alternative histones relative to canonical H3 on genes that are inducible (n = 972), expressed highly (n = 323), or weakly/not (n = 2846) in the adult head (expressional data derives from FlyBase). Statistical analysis method used: Kruskal–Wallis test. (**b**) Changes in the ratio of reads on Hsp genes relative to the total read count of the given sample (n = 13) Statistical analysis method used: Friedman-test. (**c**) Changes in the alternative/canonical histone ratio (n = 13). Statistical analysis method used: Friedman-test. n.s.: not significant; *: p < 0.05; **: p < 0.01; ***: p < 0.001; ****: p < 0.0001. Error bars represent s.d.

Interestingly, out of 2297 genes enriched in H4r and from the 2294 enriched with H3.3, 1479 genes were identical. According to expressional data of FlyBase RNA-seq expression profile, most genes showing H4r and H3.3 localization are highly expressed in the larval central nervous system and in adult brain. We found that the amount of H4r and H3.3 relative to that of the canonical H3 is significantly higher on genes that are inducible or highly expressed in the adult brain than on those genes that are weakly or not expressed (Fig. 6a). H4r/H3 ratio was 1.833 at inducible genes, 1.907 at highly expressed genes and 0.82 at weakly or not expressed genes; H3.3/H3 ratio was 1.667 at inducible genes, 1.859 at highly expressed genes and 0.937 at weakly or not expressed genes. The difference was not significant in case of the H4r/H3 ratio between the inducible and highly expressed genes (p > 0.9999) but it was significant between inducible or highly expressed genes and weakly/not expressed genes (p < 0.0001). The differences in the H3.3/H3 ratios were significant between the inducible and highly expressed genes (p = 0.0147), and between highly or inducible expressed genes and weakly/not expressed genes (p < 0.0001).

### On genes showing enrichment for H4r and H3.3 the amount of these variants changes differently upon heat stress and recovery

Increased resistance to heat stress of *H4r* knock-out flies have been described recently^5^, and we found H4r enrichment on genes involved in environmental stress responses. These observations prompted us to examine how H4r localization changes upon activation of the heat stress response and after a period of recovery time following that. We performed ChIP-seq experiments using chromatin samples obtained from heads of flies exposed to heat stress at 37 °C for 20 min and from heads of animals, which were allowed to a 3 h recovery at 25 °C after an identical heat stress. The changes in the amount of H4r and H3.3 on the Hsp genes upon heat shock and following recovery are shown on Fig. 6b, c. The level of H4r and H3.3 decreased, however, not significantly upon heat shock (p = 0.1266 and p = 0.1662, respectively), and increased significantly upon recovery (p < 0.0001 and p = 0.0006, respectively). The ratio of H4r to canonical H3 visibly increases upon heat shock (p = 0.0069) and shows a mild, but not significant increase after recovery as well (p > 0.9999). In contrast to H4r, although the ratio of H3.3 to canonical H3 increases upon heat shock (p < 0.0001), it mildly decreases after recovery (p = 0.1266). The results show a similar distribution for the two examined alternative histones upon transcription activation of Hsp genes, but indicate a difference in their distribution upon transcription silencing following the activation. According this result, nucleosomes built in during and following recovery contain more canonical H3 than H3.3, but the increasing H4r/H3 ratio suggests that these nucleosomes contain mostly H4r.

## Discussion

In this study we created an experimental system in which the alternative histone H4r and canonical H4 can be distinguished despite the same amino acid sequences they have. We used this experimental tool to examine the expression and genomic localization of H4r and to compare it to another alternative histone, to the more extensively studied H3.3. In previous studies based on RNA expression of *H4r* and deletion mutant phenotype analysis, two hypothesis arose about the function of *H4r*. One of these states that H4r replaces canonical H4 in the post-mitotic cells where canonical H4 is not expressed, and the other hypothesis states that H4r may play role in environmental stress responses.

Here we demonstrated that H4r is present already in the pronuclei of the fertilized embryos, which means that H4r is transferred to the eggs as a maternal gene product. The presence of H4r remains ubiquitous during the process of embryogenesis, and becomes to some extent cell type-specific upon neuronal differentiation. H4r differs from the H3.3 histone variant in its presence in nuclei during early embryogenesis as the latter has been described to be absent from the maternal pronucleus^7^. This might indicate function(s) which are not shared by the two alternative histones. In most mitotic cell types, on the other hand, both H3.3 and H4r are present together with their canonical histone forms. Staining of adult and larval brains for H4r revealed that it is not ubiquitously expressed in all neurons (Fig. 2 and Supplementary Figure S2). Results of ChIP-seq experiments performed with Flag-antibody specific for the 3xFlag-tagged H4r showed that the distribution of H4r throughout the genome is more similar to the other examined variant histone H3.3 than to the canonical histone H3. Both histone variants were detected preferentially bound to promoter regions of genes whereas canonical histone H3 was more abundant in intergenic regions. These findings support the assumption that H4r has a specific histone variant function. Moreover, compared to canonical H3 both histone variants were significantly more abundant on genes that are highly expressed or inducible than on weakly expressed and constitutively inactive genes. On induced Hsp gene promoters, similarly to H3.3, the amount of H4r increases upon heat shock relative to canonical H3. This notion supports the assumption that these histone variants play role in/during transcription activation. H3.3 has already been described to be involved in transcription activation^8^. H4r seems to behave similarly upon heat induction. While the amount of promoter localized H4r decreases upon heat shock, the ratio of nucleosomes that contain H4r increases, as it is seen in the case of H3.3 as well. We noticed that following activation, during recovery when transcription on Hsp genes is silenced, some H3.3 remains incorporated in nucleosomes at Hsp gene promoters but most of the newly integrated nucleosomes contain canonical H3 (Fig. 6c). H4r remains incorporated in newly inbuilt nucleosomes with higher frequency. It has been shown that H3.3 expression increased upon heat shock and most of H3.3 got incorporated into the chromatin, but during recovery H3.3 association to chromatin decreased^9^. Interestingly, *H4r* gene is not induced during heat stress and the level present in the cells is sufficient to fulfill H4 replacement connected to transcription activation of Hsp genes. Taken together these findings indicate that H4r and H3.3 may be involved in the transcription activation of inducible genes and these alternative histones might play role in the establishment of transcriptional memory. Nucleosomes containing alternative histones around promoter regions are a characteristic of inducible genes^10^. It is believed that these nucleosomes provide higher flexibility to the chromatin structure around promoters of inducible genes allowing a quicker and stronger expression upon stimulus. Our findings that H4r is abundant at promoters of genes that are inducible or associated with developmental processes and gets incorporated to promoters of Hsp genes after recovery from heat stress give rise to the assumption that H4r might play a role in establishing a transcriptional memory. Incorporation of H4r to the promoters of inducible genes might also be part of a priming for easier transcription.

*H4r* loss of function mutants show reduced viability whereas the fertility and longevity of mutants is not affected. The lack of H4r leads to minor changes in the transcriptional pattern, mostly upregulating genes^5^. Our results of H4r localization on genes associated with developmental processes and genes implicated in response to various stimuli, might be interpreted as sign of a possible role of the H4r gene to provide a H4 pool that can be involved in dynamic alteration of chromatin structure after transcriptional changes that emerges with differentiation and environmental stimuli. In differentiating cells, upon entry into G0 phase, genes associated with the differentiated state are transcribed, whereas genes that drive the cell cycle remain silenced, packed by canonical histones. In contrast, genes associated with differentiation are packed in nucleosomes by histone variants, which have replication-independent expression and provide easier access for further transcription. In the absence of H4r, maintenance of the normal transcription pattern in differentiated cells could be impaired, leading to developmental defects and consequently reduced viability.

## Materials and methods

### Generation of transgenic fly lines

We used CRISPR/Cas9 system for generation of flies expressing H4r fused with a 3xFlag tag. We followed the protocol described by Port et al., 2014^11^ and Henn et al., 2020^12^. For the guide-RNA sequences cloned into pCFD4 plasmid see Supplementary Table S2.

For modifying the *H4r* locus we amplified an extended H4r genomic region (primers used: H4r extended genomic region Fw and Rev, see Supplementary Table S2), and ligated the NheI-digested amplicon to pBlueScript II KS ( +) plasmid, creating donor plasmids for the CRIPSR/Cas9 system mediated homologous recombination. For tagging H4r, we created a subclone by amplifying the gene using H4r subcloning Fw and Rev primers, and cloning it to a modified pBlueScript II KS ( +) plasmid via BglII and KpnI. We fused 3xFlag-tag to the *H4r* gene via PCR using N-terminal Flag-tag Fw and Rev primers, generating a daughter plasmid. We made the generated daughter plasmid circularised by digesting both end with ClaI, and ligated the digested ends by T4 ligase. We cloned the tagged *H4r* gene to the donor plasmid using HpaI and BglII restriction endonuclases, generating a daughter donor plasmids. Then a new subclone plasmids was generated by cloning the modified *H4r* gene and the adjacent 265 bp upstream and 430 bp downstream sequences using SacII and PstI enzymes. On this subclone, the PAM sequences were mutated by Sequence and Ligation Independent Cloning (SLIC)^13^: the amplification of *H4r* gene surrounded by mutated PAM sequences was made by the PAM mutation insert Fw and Rev primers, the plasmid sequences bounded by the mutated PAM sequences were amplified with the PAM mutation plasmid Fw and Rev. The loxP-dsRed-loxP (derived from pHD-dsRed plasmid) sequence was cloned to the NdeI recognition sequence of the plasmids after blunting. The modified *H4r* gene and dsRed marker gene surrounded by loxP sequences were cloned to the donor plasmid by using SacII and PstI enzymes. For the sequences of the primers used see Supplementary Table S2. An outline of the cloning steps for creating donor plasmids is shown on Supplementary Figure S1.

For generation of a line expressing 3xFlag-tagged H4r and dsRed marker, we injected the donor plasmids as it is described by Henn et al., 2020^12^.

For immunohistochemical staining we created a derivative of the above described line expressing 3xFlag-H4r and dsRed. For knocking out dsRed in order to avoid high background on immunohistochemistry samples, we used Cre-mediated recombination of loxP sites.

Transgenic line carrying *UAS-H3.3-3xFlag* gene (*w; UAS-H3.3-3xFlag*)^14^ was crossed with *elav-Gal4* (BL458) for ChIP-seq experiments. Chromatin samples were prepared from the heads of adult offspring of this crossing.

### Immunohistochemistry and western blotting

Immunostaining of embryos was performed as described by Henn et al.,^12^. Larval and adult brains were dissected in Ringer’s solution and then fixed in PBS containing 4% formaldehyde by rotating for 20 min at room temperature. After removing the fixative solution, samples were washed three times for 5 min at room temperature in PBT (0.1% Triton-X-100 in PBS). They were blocked in PBT-N (0.1% Triton-X-100, 1% BSA and 5% FBS in PBS) at room temperature for 1 h. Samples were incubated overnight in PBT-N containing primary antibodies, then they were washed three times at room temperature in PBT for 10 min, then incubated in PBT-N containing secondary antibodies and DAPI for 1 h at room temperature. After another three washes with 10 min of PBT, samples were placed on microscope slide and mounted in Fluoromount-G (Invitrogen). For positive and negative controls of the immunohistochemical experiments see Supplementary Figure S3.

To prepare complete protein extracts for western blot 15 adult heads were homogenized in RIPA buffer (150.4 mM NaCl; 0.1% SDS; 0.5% Na-DOC; 0.01% Triton-X-100; 1 × PIC; 0.025 M Tris–HCl pH 7.5) (4 μl/adult head) and then incubated on ice for 30 min. At the end of incubation time, samples were centrifuged at 13,000 rpm for 10 min, and the supernatants were transferred to clean tubes. Pellets were resuspended in an amount of RIPA buffer equal to the amount of supernatants, and the supernatants containing the soluble proteins and the resuspended pellets containing the chromatin-bound proteins were boiled with SDS Loading buffer for 10 min.

Proteins were separated via Tricin-SDS-PAGE^15^. Prior to blotting, membranes were washed in methanol for 15 s, then in water for 2 min, then in blotting buffer (0.02 M Tris–HCl pH 8.0; 0.15 M glycine; 20% methanol). Following blotting, membranes were blocked in TBST (10 mM Tris–HCl pH 8.0; 150 mM NaCl; 0.05% Tween-20) containing 5% non-fat dry milk for 1 h at room temperature, then membranes were incubated in TBST containing 0.02% BSA and primary antibodies at 4 °C overnight. After removing TBST with BSA and primary antibodies, membranes were washed in TBST four times for 10 min and then they were incubated in TBST containing 0.02% BSA and secondary antibodies for 1 h at room temperature. After washing them four times for 10 min in TBST, membranes were incubated in 10 × diluted ECL reagent (Millipore) at room temperature for 5 min then signals were detected via Li-Cor C-DiGit Scanner and measured with ImageJ software. For statistical analysis one-way ANOVA with Tukey’s multiple comparison test was performed via GraphPad Prism 8.0.1.

Antibodies used for IHC and WB: mouse α-Flag (Sigma M2) in 1:1000 dilution for IHC and 1:5000 for WB; rabbit α-GFP (A-6455) in 1:500 dilution for IHC, rabbit α-H4 (ab10158) in 1:1000 dilution for WB, goat anti-mouse Alexa Fluor 488 (ab150113) in 1:600 dilution, donkey anti-rabbit Alexa Fluor 555 (ab150074) in 1:600 dilution, goat anti-mouse Alexa Fluor 568 (Ab175473) in 1:600 dilution, DAPI in 1:500 dilution, rabbit anti-mouse/HRP (Dako, P0260) in 1:10,000 dilution, goat anti-rabbit/HRP (Dako, P0448) in 1:10,000 dilution. Larval and adult brains stained with only a-Flag antibody and DAPI were visualized with spinning disk confocal microscope (Visitron spinning disk confocal microscope with Yokogawa CSU-W1 unit and Andor Zyla 4.2 PLUS sCMOS camera) using 20 × dry objective (NA: 0.45), composite images were prepared using ImageJ software. Embryos and larval brains stained with a-Flag, a-GFP antibodies and DAPI were visualized with Leica SP5 AOBS confocal laser scanning microscope with 20 × dry (NA: 0.7) objective, composite images and co-localization ratio counting were performed using Leica LAS AF Software.

### RNA extraction and qPCR

RNA was extracted from adult heads homogenized in NE buffer (15 mM HEPES pH 7.6, 10 mM KCl, 5 mM MgCl_2_, 0.1 mM EDTA, 0.5 mM EGTA, 350 mM sucrose, 0.1% Tween 20, 1 mM DTT, 1 × PIC (Proteinase Inhibitor Cocktail (Roche))). RNA extraction was performed by using TRIzol Reagent (Thermo) according the recommendations of the manufacturer. DNA contamination in the extracted RNA samples were removed with DNaseI (Thermo), and reverse transcription was performed via TaqMan Reverse Transcription Reagents (Invitrogen). qPCR was performed by using Promega GoTaq qPCR Master Mix (Thermo). The primers used for qPCR are shown in Supplementary Table S2. For statistical analysis one-way ANOVA with Sidak’s multiple comparison test was performed via GraphPad Prism 8.0.1.

### Chromatin-immunoprecipitation and sequencing

Chromatin-immunoprecipitation was performed as described by Schauer et al., 2013^16^, with the following modifications: 0.954–2.575 µg chromatin was used to each ChIP assay. Dynabeads protein G (Invitrogen) matrix was equilibrated in RIPA buffer without carrier DNA and BSA. After immunopurification, beads were washed five times with RIPA buffer, once with LiCl buffer and once with 10 mM Tris–HCl pH 8.0 (without EDTA), 5 min ea. Beads were resuspended in 10 mM Tris–HCl pH 8.0. Samples were incubated with Proteinase K (Serva) for 3 h at 50 °C after removing RNA contamination and reverse crosslinking. Immunoprecipitated DNA was purified by phenol–chloroform-isoamilalcohol extraction followed by precipitation with ethanol. DNA samples were resuspended in 10 mM Tris–HCl pH 8.0.

Library preparation was performed by using NEBNext Ultra II DNA Library Prep Kit for Illumina according the recommendations of the manufacturer, without size selection. MiSeq Reagent Kit v3 was used for sequencing. For each sample, two biological replicates were sequenced two times.

### Sequence alignment, peak calling, read counting and gene ontology annotation

Reads were trimmed with Trim Galore (0.6.6.) (https://github.com/FelixKrueger/TrimGalore) and aligned to the *Drosophila melanogaster* (dm6) genome with Bowtie2 (2.4.2) software^17^. Blacklisted^18^ reads were removed via BAM Filter Galaxy Version 0.5.9 software^19^. BAM files of replicates of samples were merged via Merge BAM Files (Galaxy Version 1.2.0.) (https://gatk.broadinstitute.org/hc/en-us/articles/360036485412-MergeSamFiles-Picard-). Correlation matrix comparing samples were generated by using multiBamSummary Galaxy Version 3.3.2.0.0.0 software^20^, scatterplots were made with Spearman’s correlation method via plotCorrelation Galaxy Version 3.3.2.0.0^20^. Peak calling were performed on the filtered BAM files via MACS2 callpeak Galaxy Version 2.1.1.20160309.6^21,22^, peaks were annotated via ChIPseeker Galaxy Version 1.18.0 + galaxy1 software^23^. The differences in peaks between samples were calculated by a MACS2 bdgdiff Galaxy Version 2.1.1.20160309.1^21,22^. Since total input controls were not sequenced, peaks and reads of variant histones were compared with normalization to H3 reads of the given samples. Genomic annotations were performed on *Drosophila melanogaster* assembly release BDGP6.28.101. Read numbers were determined using BedCov Galaxy Version 2.0.2^24^, and read numbers were normalized following this formula: number of reads on a given region in a given sample / total number of reads in a given sample. For determining read counts on entire genes, annotation file was downloaded from FlyMine (https://www.flymine.org/flymine/regions). For gene ontology annotations PANTHER GO-Slim (http://pantherdb.org/) with Fisher’s Exact test type and FDR correction was used. Expressional data for counting reads on highly, inducible or weakly/not expressed genes derive from FlyBase expression profile (http://flybase.org/rnaseq/profile_search): highly expressed genes are determined as genes showing peak expression levels not less than ‘very high’ in adults in the age of 1–5 days and peak expression levels not less than ‘moderately high’ in adult heads. Weakly or not expressed genes are determined as genes showing peak expression levels not higher than ‘low’ in adults in the age of 1–5 days and peak expression levels not higher than ‘no/extremely low’ in adult heads. Inducible genes are determined as genes showing peak expression levels not less than ‘very high’ in adults in the age of 1–5 days and peak expression level not less than ‘moderately high’ in adult heads in case of any treatment with available expressional data. Statistical analysis of the comparisons for the genome-wide distribution of H4r, H3.3 and H3 was performed using two-way ANOVA with Tukey’s multiple comparison test was performed via GraphPad Prism 8.0.1. For the statistical analysis of H4r and H3.3 abundance relative to H3 on inducible, highly and weakly/not expressed genes Kruskal–Wallis test followed with Dunn’s multiple comparison test was used via GraphPad Prism 8.0.1. For statistical analysis of the changes in the absolute and relative abundance of H4r and H3.3 on the Hsp genes in the distinct conditions Friedman-test followed with Dunn’s multiple comparison test was used via GraphPad Prism 8.0.1.

## Supplementary Information

Supplementary Information 1.

Supplementary Information 2.

Supplementary Information 3.

Supplementary Information 4.

Supplementary Information 5.

## Data Availability

Datasets generated and used in this study are available on the National Center for Biotechnology Information Sequence Read Archive (NCBI SRA) under accession GSE197256.

## References

[1] Yamamoto Y, Watanabe T, Matsuo Y (2016). Epigenetics evolution and replacement histones: Evolutionary changes at Drosophila H4r. J. Phylogenet. Evol. Biol..

[2] Akhmanova A, Miedema K, Hennig W (1996). Identification and characterization of the Drosophila histone H4 replacement gene. FEBS Lett..

[3] Morozova TV, Mackay TFC, Anholt RRH (2011). Transcriptional networks for alcohol sensitivity in Drosophila melanogaster. Genetics.

[4] Copur Ö, Gorchakov A, Finkl K, Kuroda MI, Müller J (2018). Sex-specific phenotypes of histone H4 point mutants establish dosage compensation as the critical function of H4K16 acetylation in Drosophila. PNAS.

[5] Faragó A, Ürmösi A, Farkas A, Bodai L (2021). The histone replacement gene His4r is involved in heat stress induced chromatin rearrangement. Sci. Rep..

[6] Marshall OJ, Brand AH (2017). Chromatin state changes during neural development revealed by in vivo cell-type specific profiling. Nat. Commun..

[7] Konev AY (2007). CHD1 motor protein is required for deposition of histone variant H3.3 into chromatin in vivo. Science.

[8] Sakai A, Schwartz BE, Goldstein S, Ahmad K (2009). Transcriptional and developmental functions of the H3.3 histone variant in Drosophila. Curr Biol.

[9] Schwartz BE, Ahmad K (2005). Transcriptional activation triggers deposition and removal of the histone variant H3.3. Genes Dev..

[10] Zhou M (2021). Structural basis of nucleosome dynamics modulation by histone variants H2A.B and H2A.Z.2.2. EMBO J.

[11] Port F, Chen H-M, Lee T, Bullock SL (2014). Optimized CRISPR/Cas tools for efficient germline and somatic genome engineering in Drosophila. Proc. Natl. Acad. Sci. U. S. A..

[12] Henn L (2020). Alternative linker histone permits fast paced nuclear divisions in early Drosophila embryo. Nucleic Acids Res..

[13] Jeong J-Y (2012). One-step sequence- and ligation-independent cloning as a rapid and versatile cloning method for functional genomics studies. Appl. Environ. Microbiol..

[14] Song W, Zsindely N, Faragó A, Marsh JL, Bodai L (2018). Systematic genetic interaction studies identify histone demethylase Utx as potential target for ameliorating Huntington’s disease. Hum. Mol. Genet..

[15] Schägger H (2006). Tricine-SDS-PAGE. Nat. Protoc..

[16] Schauer T (2013). CAST-ChIP maps cell-type-specific chromatin states in the Drosophila central nervous system. Cell Rep..

[17] Langmead B, Wilks C, Antonescu V, Charles R (2019). Scaling read aligners to hundreds of threads on general-purpose processors. Bioinformatics.

[18] Amemiya HM, Kundaje A, Boyle AP (2019). The ENCODE Blacklist: Identification of Problematic Regions of the Genome. Sci. Rep..

[19] Mendoza-Parra MA, Saleem M-AM, Blum M, Cholley P-E, Gronemeyer H (2016). NGS-QC generator: A quality control system for ChIP-Seq and related deep sequencing-generated datasets. Methods Mol. Biol..

[20] Ramírez F (2016). deepTools2: A next generation web server for deep-sequencing data analysis. Nucleic Acids Res..

[21] Zhang Y (2008). Model-based analysis of ChIP-Seq (MACS). Genome Biol..

[22] Feng J, Liu T, Qin B, Zhang Y, Liu XS (2012). Identifying ChIP-seq enrichment using MACS. Nat. Protoc..

[23] Yu G, Wang L-G, He Q-Y (2015). ChIPseeker: An R/Bioconductor package for ChIP peak annotation, comparison and visualization. Bioinformatics (Oxford, England).

[24] Li H (2009). The sequence alignment/map format and SAMtools. Bioinformatics.

[25] Mahr A, Aberle H (2006). The expression pattern of the Drosophila vesicular glutamate transporter: A marker protein for motoneurons and glutamatergic centers in the brain. Gene Expr. Patterns.

[26] Nässel DR, Enell LE, Santos JG, Wegener C, Johard HA (2008). A large population of diverse neurons in the Drosophilacentral nervous system expresses short neuropeptide F, suggesting multiple distributed peptide functions. BMC Neurosci..

[27] Yasuyama K, Meinertzhagen IA, Schürmann F-W (2002). Synaptic organization of the mushroom body calyx in Drosophila melanogaster. J. Comp. Neurol..

[28] Kraut R, Campos-Ortega JA (1996). inscuteable, A neural precursor gene of Drosophila, encodes a candidate for a cytoskeleton adaptor protein. Dev. Biol..

[29] Robinow S, White K (1991). Characterization and spatial distribution of the ELAV protein during Drosophila melanogaster development. J. Neurobiol..

