# Supplementary material for: Despite its sequence identity with canonical H4, Drosophila H4r product is enriched at specific chromatin regions
Source: Sci Rep. 2022 Mar 23;12:5007. doi: 10.1038/s41598-022-09026-x (PMC8943024; doi:10.1038/s41598-022-09026-x)
Supplement: Supplementary file 1 — Supplementary Information 1. [file 41598_2022_9026_MOESM1_ESM.pdf]

Supplementary Figures

A

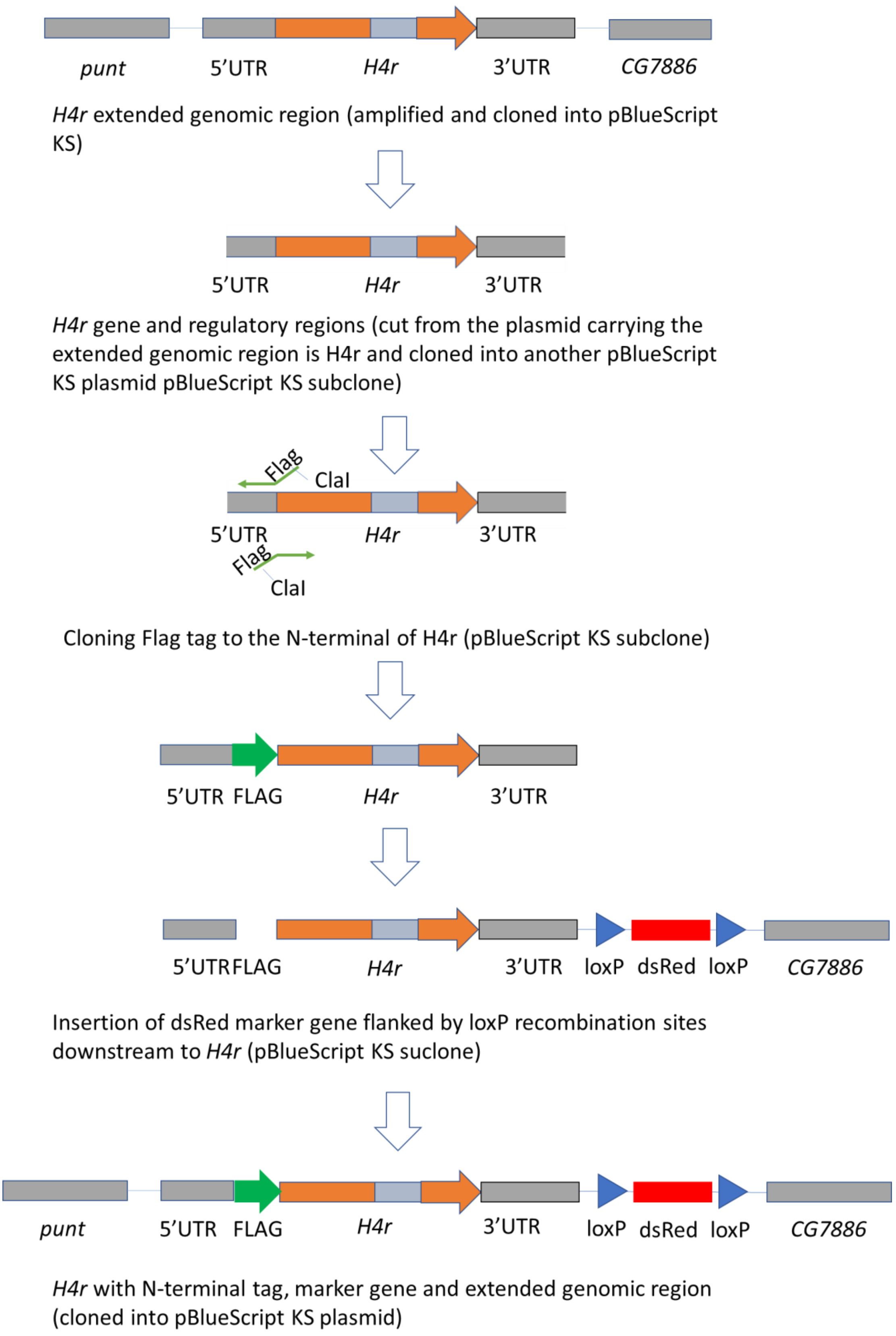

B

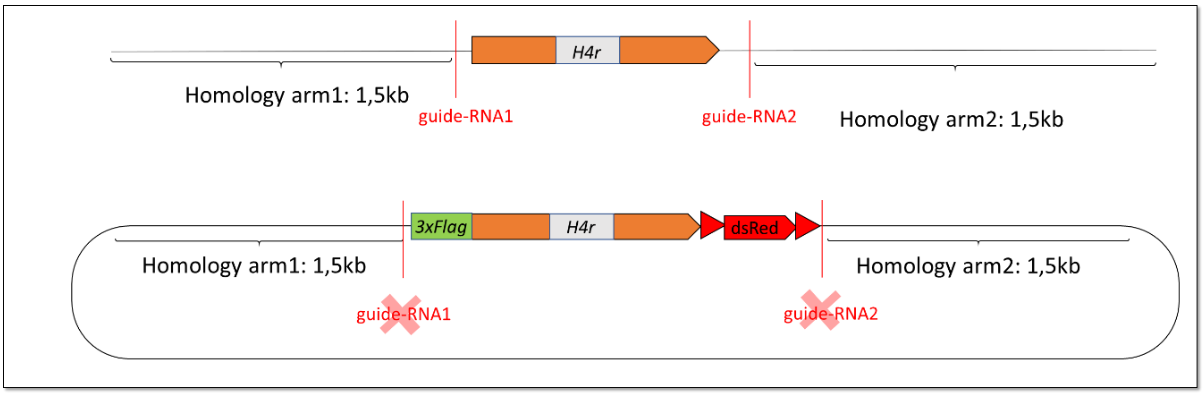

Figure S1: Strategy used for *in-situ* modification of the *H4r* gene via CRISPR/Cas9 system. A: Outline of the used cloning scheme for generation of donor plasmids to exchange *H4r* gene with 3xFlag-H4r via CRISPR/Cas9. B: Exchanging the *H4r* gene with a 3xFlag-tagged one carrying a downstream *dsRed* marker gene. guide-RNA1 sequence: CTGTAAAGCGACGCCATGTT; guide-RNA2 sequence: GGCAGTAGCAGTAGAAAAGGC.

a *w; ChAT-Gal4/UAS-Lam-GFP; 3xFlag-His4r*

Lam-GFP **Flag (His4r)** **DAPI** **merge**

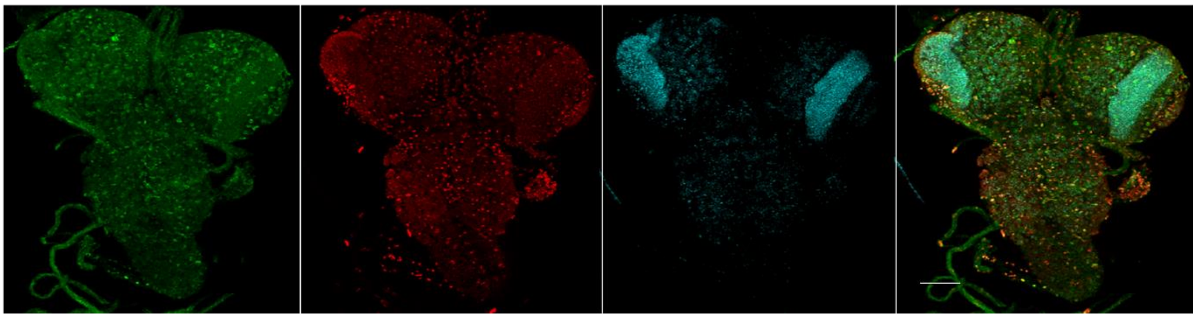

b *elav-Gal4/w; UAS-Lam-GFP/+; 3xFlag-His4r/+*

elav-Gal4/Y; UAS-Lam-GFP/+; 3xFlag-His4r/+

Lam-GFP **Flag (His4r)** **DAPI** **merge**

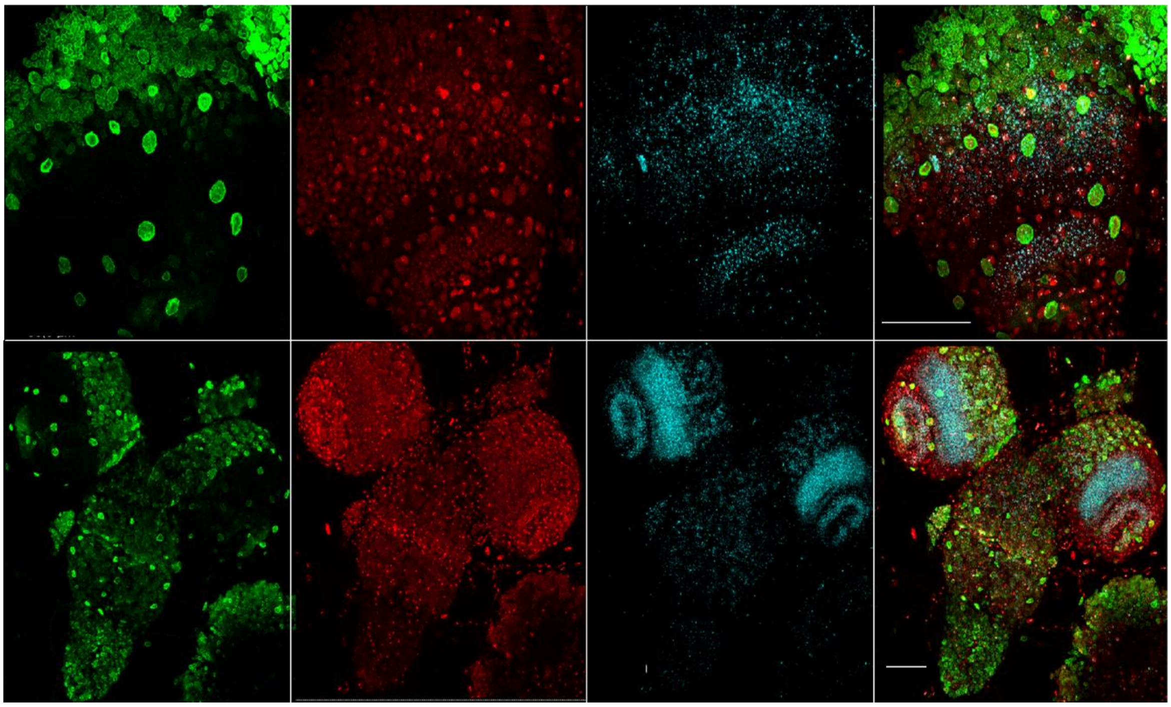

c *w; OK371-Gal4/UAS-Lam-GFP; 3xFlag-His4r*

Lam-GFP **Flag (His4r)** **DAPI** **merge**

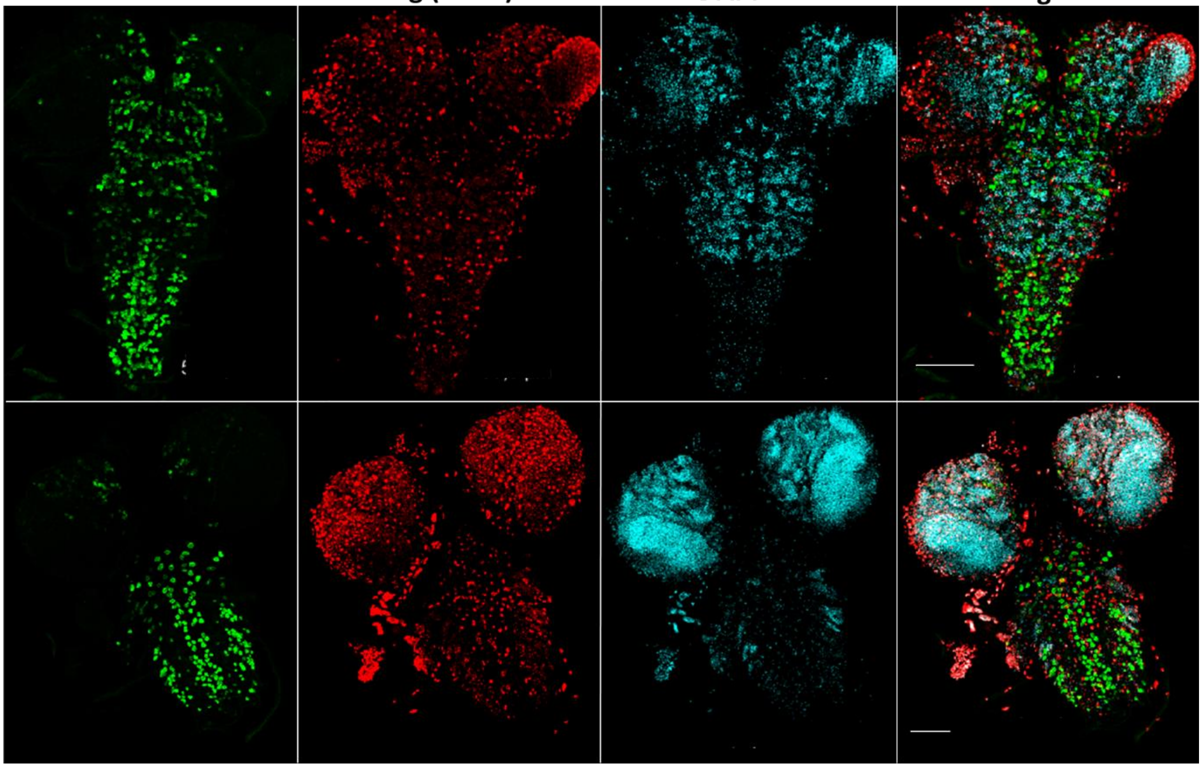

d *w; Gad1-Gal4/UAS-Lam-GFP; 3xFlag-His4r*

Lam-GFP **Flag (His4r)** **DAPI** **merge**

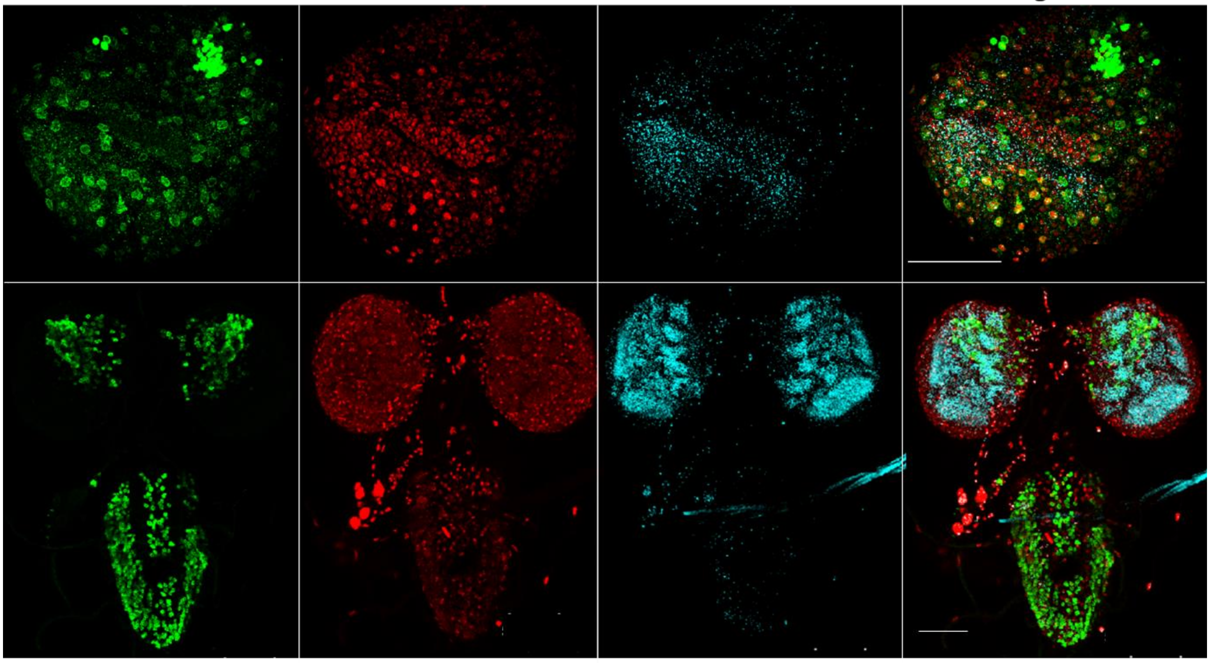

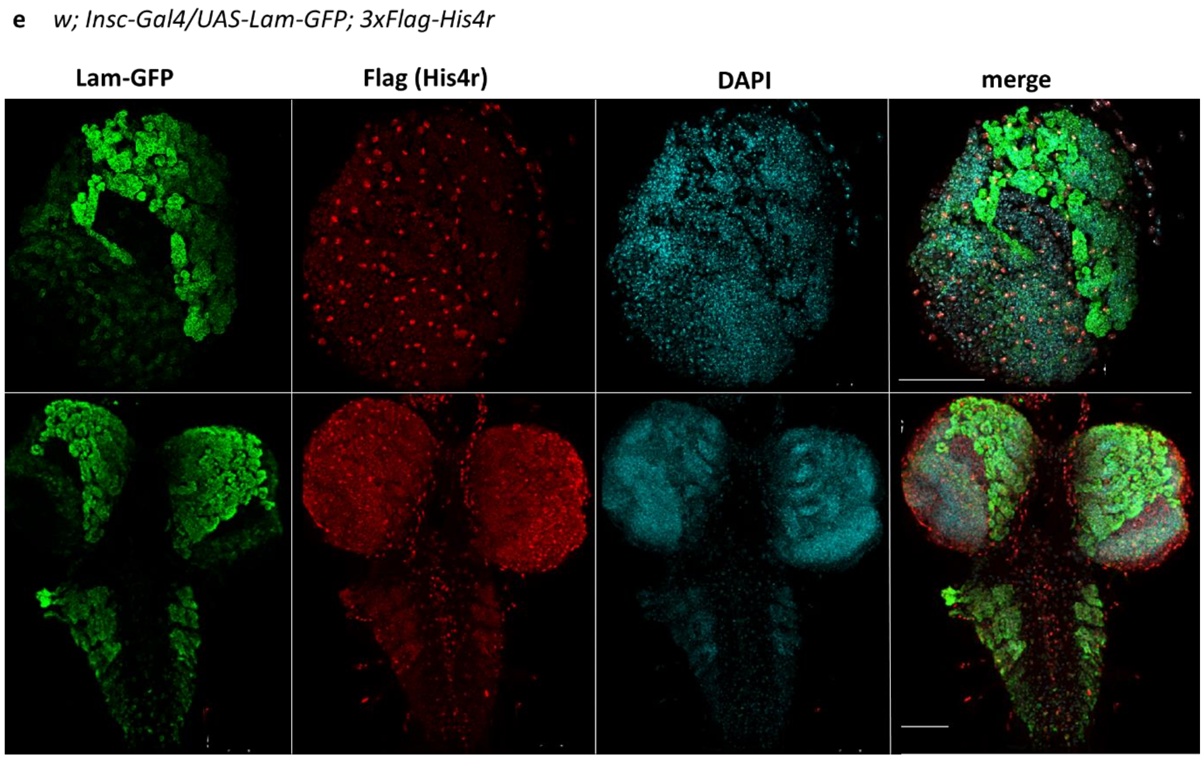

Figure S2: Weak co-localization between His4r and Lam-GFP expressed under the control of different neuronal type-specific Gal4-drivers. Lam-GFP expressed in **a:** cholinergic neurons, **b:** mature neurons, **c:** glutaminergic neurons, **d:** GABAergic neurons, **e:** neuroblasts. Up: eye-disc, Down: entire larval brain. Scale bars refer to 50 µm.

a *w^{1118} (negative control)*

Lam-GFP **Flag (His4r)** **DAPI** **merge**

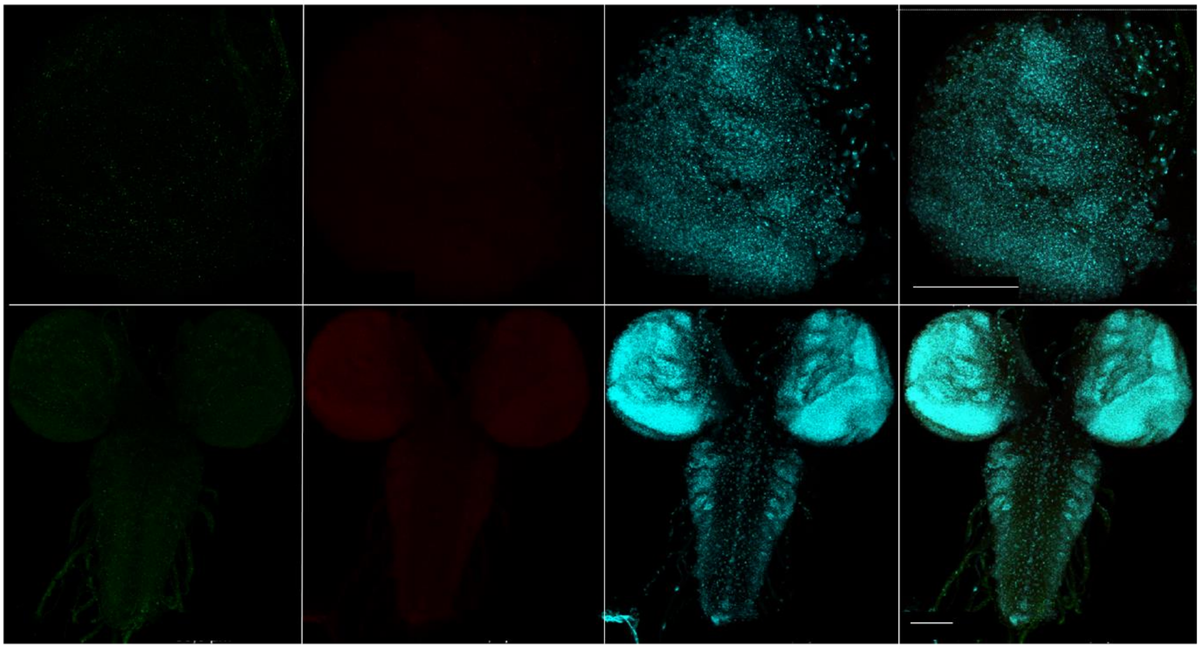

b *w; Act5C-Gal4/+; UAS-His4r-3xFlag/+ (positive control)*

Lam-GFP **Flag (His4r)** **DAPI** **merge**

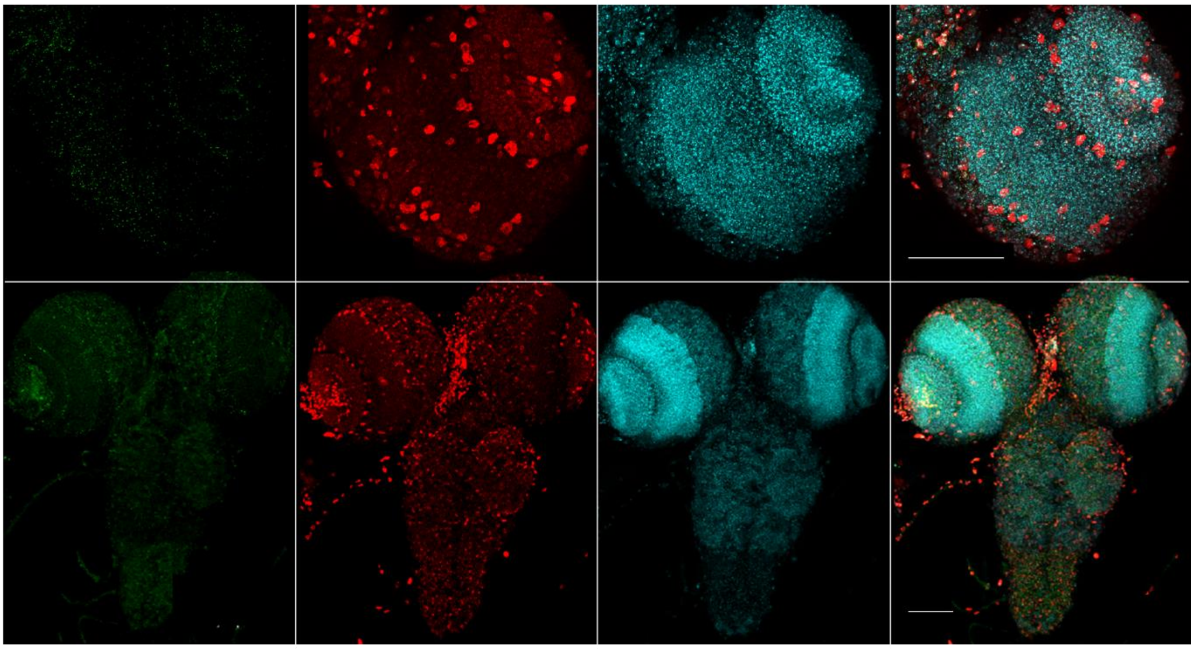

c **Flag** **DAPI** **merge**

w^{1118} (negative control)

w; 3xFlag-BigH1^{dsRed} (positive control)

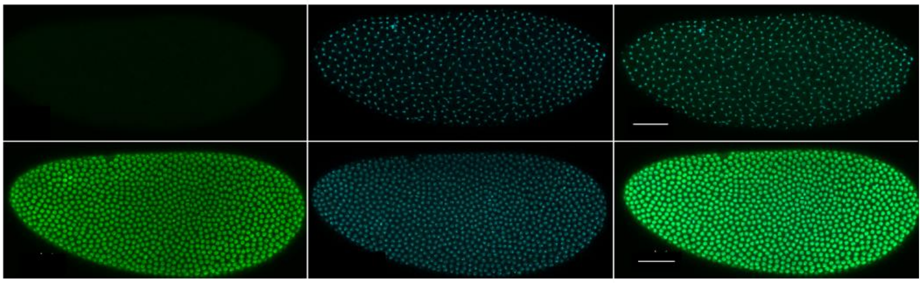

d

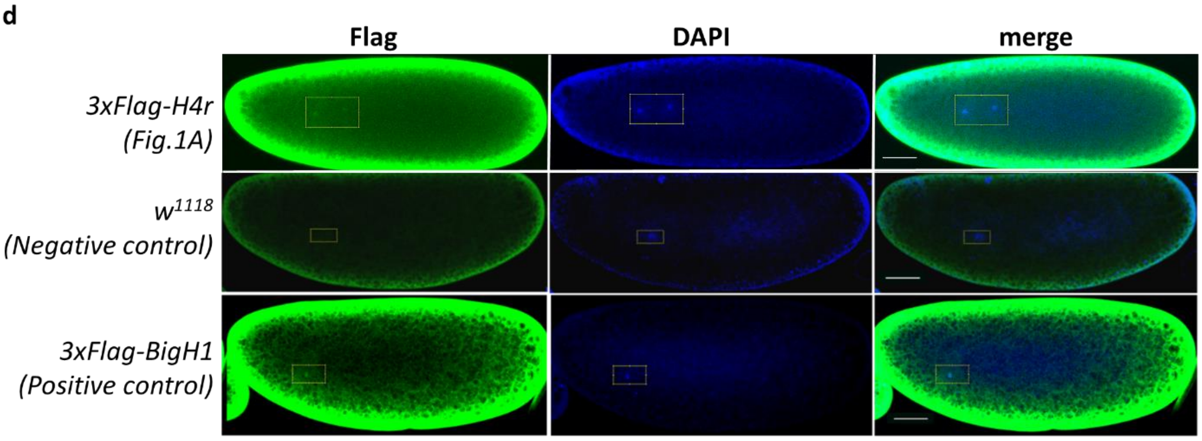

Figure S3: Controls of Flag-stainings in immunohistochemical assays. A: negative control in larval brains; **B:** positive control in larval brains. Up: eye-discs; Down: entire larval brains. **C:** controls in embryos. Up: negative control; Down: positive control. **D:** controls in embryos with pronuclei. Up: negative control; Down: positive control[12]. Scalebars refer to 50 µm.

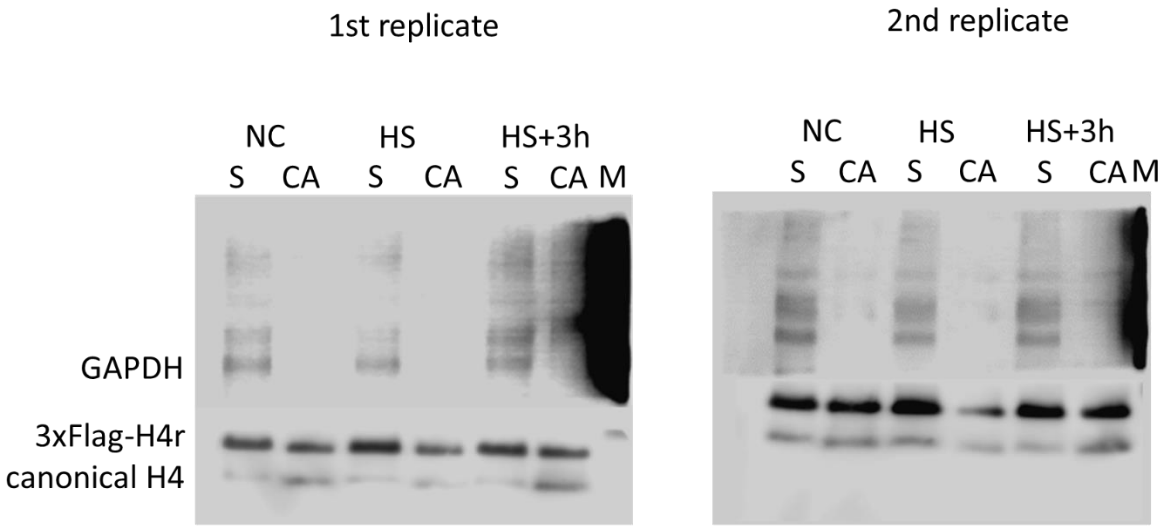

Figure S4: Uncropped western blot images part of which were used as Figure 3. For both gels shown following transfer the membranes were cut at a position corresponding to the migration of a 25 kDa protein and the two parts were developed with GAPDH and Flag plus H4 Ab, respectively. Since the used GAPDH Ab was not satisfactorily specific the upper parts of the images were not used to draw conclusions on expression changes. NC: negative control; HS: heat shocked; HS+3h: heat shocked and relaxed (at room temperature) for three hours. S: soluble, CA: chromatin associated, M: Molecular weight protein ladder (Thermo).
