# Supplementary material for: Despite its sequence identity with canonical H4, Drosophila H4r product is enriched at specific chromatin regions
Source: Sci Rep. 2022 Mar 23;12:5007. doi: 10.1038/s41598-022-09026-x (PMC8943024; doi:10.1038/s41598-022-09026-x)
Supplement: Supplementary file 2 — Supplementary Information 2. [file 41598_2022_9026_MOESM2_ESM.docx]

**Table S1: Gene ontology annotations of ChIP-seq data.** ChIP-seq results obtained from H4r and H3.3 IPs are annotated as follows: first table (from the left): genes showing H4r enrichment; second table: genes showing H3.3 enrichment; third table: genes showing both H4r and H3.3 enrichment; fourth table: genes showing H4r but no H3.3 enrichment; fifth table: genes showing H3.3 but no H4r enrichment.
