# Supplementary material for: Despite its sequence identity with canonical H4, Drosophila H4r product is enriched at specific chromatin regions
Source: Sci Rep. 2022 Mar 23;12:5007. doi: 10.1038/s41598-022-09026-x (PMC8943024; doi:10.1038/s41598-022-09026-x)
Supplement: Supplementary file 4 — Supplementary Information 4. [file 41598_2022_9026_MOESM4_ESM.docx]

| guide-RNA cloning Fw: | ATATAGGAAAGATATCCGGGTGAACTTCGCTGTAAAGCGACGCCATGTTGTTTTAGAGCTAGAAATAGCAAG |
| --- | --- |
| guide-RNA cloning Rev: | ATTTTAACTTGCTATTTCTAGCTCTAAAACGGCAGTAGCAGTAGAAAAGGCGACGTTAAATTGAAAATAGGTC |
| H4r extended genomic region Fw: | AGATACGCTAGCAGGCACATTATGCACACAGAAAGGGATG |
| H4r extended genomic region Rev: | ATGGTTCA AGAGAAGGCCATGGAGGGAGG |
| H4r subcloning Fw: | GCAAACTTGTTTACAGCACTCGTTGCG |
| H4r subcloning Rev: | TGCGTTCGGTACCGTT TGCGTTGAATACTACTGATTAAG |
| N-terminal Flag-tag Fw: | CATGACATCGATTACAAGGATGACGATGACAAGATGACTGGTCGTGGAAAGGGAGGCAAAGG |
| N-terminal Flag-tag Rev: | CTTGTAATCGATGTCATGATCTTTATAATCACCGTCATGGTCTTTGTAGTCCATTTCTCAGTTGCTTCGTAAAGTTGGCTG |
| PAM mutation insert Fw: | TTACCTTTCATCAAACATGGCGTCG |
| PAM mutation insert Rev: | AATTTGCAGTCATACTCACGGGC |
| PAM mutation plasmid Fw: | CTGCCCGTGAGTATGACTGC |
| PAM mutation plasmid Rev: | CGACGCCATGTTTGATGAAAGG |
| qH4r Fw: | TTATAAAGATCATGACATCGATTACAAGGATGACGA |
| qH4r Rev: | CGAGTTTCCTCGTAAATCAAGCCAGAG |
| Alpha-tubulin at 84B Fw: | TGTCGCGTGTGAAACACTTC |
| Alpha-tubulin at 84B Rev: | AGCAGGCGTTTCCAATCTG |

**Table S2: Primers used in this study.**
