# Supplementary material for: Despite its sequence identity with canonical H4, Drosophila H4r product is enriched at specific chromatin regions
Source: Sci Rep. 2022 Mar 23;12:5007. doi: 10.1038/s41598-022-09026-x (PMC8943024; doi:10.1038/s41598-022-09026-x)
Supplement: Supplementary file 5 — Supplementary Information 5. [file 41598_2022_9026_MOESM5_ESM.docx]

| Genotype | ID | Abbreviated name |
| --- | --- | --- |
| *w[1118]; P{w[+mW.hs]=GawB}VGlut[OK371]* | RRID:BDSC_26160 | OK371-Gal4 |
| *P{w[+mC]=Gad1-GAL4.3.098}2/CyO* | RRID:BDSC_51630 | Gad1-Gal4 |
| *w[1118]; P{w[+mC]=ChAT-GAL4.7.4}19B/CyO, P{ry[+t7.2]=sevRas1.V12}FK1* | RRID:BDSC_6798 | ChAT-Gal4 |
| *w[*]; P{w[+mW.hs]=GawB}insc[Mz1407]* | RRID:BDSC_8751 | Insc-Gal4 |
| *P{w[+mW.hs]=GawB}elav[C155]* | RRID:BDSC_458 | elav-Gal4 |
| *y[1] w[*]; P{w[+mC]=UAS-Lam.GFP}3-2/CyO* | RRID:BDSC_7377 | Lam-GFP |
| *y^1^v^1^P{nos-phiC31\int.NLS}X; P{CaryP}attP40* | RRID:BDSC_25709 | - |
| *y^2^cho^2^v^1^; wg^Sp-1^/CyO* | NIG-FLY TBX-0009 | - |
| *y^1^ M{Act5C-Cas9.P}ZH-2A w** | RRID:BDSC_54590 | - |
| *w;  TM3, Sb / TM6B, Hu Tb* | - | - |

**Table S3: Used *Drosophila* stocks.**
